# Remedies and Their Uses

**Published:** 1906-09-15

**Authors:** 


### Remedies and their Uses.

### Local Anæsthesia with Orthoform.

Orthoform is a valuable local anæsthetic, but it has the practical disadvantage of being somewhat expensive. The new orthoform, called by its manufacturers (Meister Lucius and Brüning, of Hœchst-a-M.) orthoform new, was introduced as a cheap substitute, and has little to differentiate it in practice from the old preparation.

Its physiological action resembles that of cocaine in so far that it paralyses all sensory nerves and nerve endings when directly applied. It does not, however, act through the unbroken skin or mucous membranes. It is also slightly antiseptic. It is, in ordinary doses, absolutely non-toxic, thus having a great advantage over cocaine.

Orthoform new has been employed as an anæsthetic in cases of gastric ulcer, gastric carcinoma, ulcerative stomatitis, and tuberculous and syphilitic affections of the pharynx and larynx. It can also be used to plug carious and painful teeth or to lessen the pain after extractions. As a snuff it has been recommended in hay fever, and as a dusting powder or ointment it has been used on cutaneous wounds and ulcers of a painful nature. A hydrochloride has also been prepared which is soluble in 10 parts of water and gives an acid reaction.

The dosage of orthoform new by the mouth is 7½-15 grains several times daily. It should be given on an empty stomach. It may be taken in half a tumbler of water, or suspended in an emulsion. In cystitis it has been used as an injection, 15-30 grains in normal saline (ʒj. to OjNaCl) and in urethritis a bougie containing 2 per cent. orthoform combined with iodoform or dermatol has been found useful. Externally, in painful ulcerating surfaces, it may be applied as an emulsion:

| | |
|---|---|
| Menthol ... ... ... ... ... ... | 10 |
| Orthoform new ... ... ... ... | 10 |
| Ol. amygd. dulc. ... ... ... ... | 30 |
| Aq. destil. ad ... ... ... ... ... | 100 |

made up with the yolks of two eggs. An ointment 10 per cent. to 20 per cent. with lanoline may be used in such conditions as pruritis vulvæ, herpes zoster, etc. In hay fever a snuff composed of

| | |
|---|---|
| Orthoform new ... ... ... ... | 1 part |
| Pulv. sacch. alb. ... ... ... ... | 4 parts |

may be ordered.

Very good results in tuberculous laryngitis have been reported by spraying the larynx with :

| | |
|---|---|
| Orthoform new ... ... ... | 5 parts |
| Menthol. ... ... ... ... | 10-20 parts |
| Ol. olivæ ad ... ... ... | 100 parts |

Ulcerated surfaces may be simply dusted over with the powdered crystals.

As a stopping for painful carious teeth the following may be used :

| | |
|---|---|
| Orthoform new ... ... ... ... | 1 part |
| Acidi carbolici ... ... ... ... | 1 part |
| Camphor. ... ... ... ... ... | 4 parts |
| Chlorol hydrat. ... ... ... ... | 4 parts |

